# Supplementary material for: Fibrolytic rumen bacteria of camel and sheep and their applications in the bioconversion of barley straw to soluble sugars for biofuel production
Source: PLoS One. 2022 Jan 7;17(1):e0262304. doi: 10.1371/journal.pone.0262304 (PMC8740978; doi:10.1371/journal.pone.0262304)
Supplement: S1 Table — (DOCX) [file pone.0262304.s001.docx]

**S1 Table: Relative abundances (%) of bacterial families and genera colonized to straw incubated with rumen inoculums of camel and sheep.**

|  | **family** | **Genus** | Camel (FC) | SE | Sheep (FR) | SE | Overall mean | SEM | P-value |
| --- | --- | --- | --- | --- | --- | --- | --- | --- | --- |
| Phylum Actinobacteria | | | | | | | | | |
| Atopobiaceae | | Olsenella |  |  | 0.28 |  |  |  |  |
| Phylum Bacteroidetes | | | 19.07 | 6.3 | 26.6 | 5.24 | 22.86 | 4.05 | P>0.05 |
| Muribaculaceae | | |  |  | 0.08 |  |  |  |  |
| Rikenellaceae | | RC9_gut_group | 18.28 | 6.1 | 26.09 | 5.2 | 22.2 | 3.99 | P>0.05 |
| Prevotellaceae | | Prevotella_1 | 0.79 | 0.25 | 0.48 | 0.2 | 0.64 | 0.16 | P<0.05 |
| Phylum Firmicutes | |  | 80.89 | 6.37 | 72.64 | 5.18 | 76.76 | 4.1 | P>0.05 |
| Ruminococcaceae | | | 24.78 | 3.3 | 18.4 | 2.44 | 21.59 | 2.3 | P>0.05 |
| Ruminococcaceae | | Ruminococcus_2 | |  | 0.14 |  |  |  |  |
| Ruminococcaceae | | Ruminococcus_1 | 14.8 | 3.2 | 9.95 | 1.34 | 12.4 | 1.9 | P>0.05 |
| Ruminococcaceae | | Saccharofermentans | 8.6 | 1.25 | 6.5 | 1.76 | 7.6 | 1.07 | P>0.05 |
| Ruminococcaceae | | UCG-010 |  |  | 0.16 |  |  |  |  |
| Ruminococcaceae | | UCG-014 | 0.14 (1n) |  | 0.42 |  |  |  |  |
| Ruminococcaceae | | Papillibacter | 0.16 |  |  |  |  |  |  |
| Ruminococcaceae | | UCG-002 |  |  | 0.16 |  |  |  |  |
| Ruminococcaceae | | NK4A214_group | 0.74 | 0.32 | 0.65 | 0.32 | 0.7 | 0.2 | P>0.05 |
| Ruminococcaceae | | UCG-004 | 0.23 | 0.16 | 0.3 | 0.03 | 0.27 | 0.07 | P>0.05 |
| Ruminococcaceae | | UCG-005 | 0.16 | 0.03 | 0.17 | 0.05 | 0.17 | 0.02 | P>0.05 |
| Lachnospiraceae | | | 30.4 | 3.2 | 37.1 | 5.3 | 33.7 | 3.12 | P>0.05 |
| Lachnospiraceae | | Pseudobutyrivibrio | 0.93 | 0.36 | 0.66 | 0.15 | 0.8 | 0.18 | P>0.05 |
| Lachnospiraceae | | Butyrivibrio_2 | 2.47 | 0.7 | 1.6 | 0.2 | 2.04 | 0.39 | P>0.05 |
| Lachnospiraceae | | probable_genus_10 | 6.09 | 1.39 | 20.4 | 8.5 | 13.26 | 5.02 | P>0.05 |
| Lachnospiraceae | | FCS020_group | 0.18 | 0.06 | 0.23 | 0.09 | 0.2 | 0.05 | P>0.05 |
| Lachnospiraceae | | Lachnoclostridium_10 | 0.7 |  | 0.46 (1n) |  |  |  |  |
| Lachnospiraceae | | FCS020_group | 8.48 | 2.4 | 7.5 | 2.14 | 8.02 | 1.47 | P>0.05 |
| Lachnospiraceae | | AC2044_group | 3.54 | 0.13 | 2.66 | 0.55 | 3.1 | 0.32 | P>0.05 |
| Lachnospiraceae | | NK4A136_group | 3.59 | 0.97 | 1.36 | 0.06 | 2.48 | 0.66 | P>0.05 |
| Lachnospiraceae | | NA | 1.01 | 0.4 | 0.87 | 0.1 | 0.94 | 0.193 | P>0.05 |
| Lachnospiraceae | | FD2005 | 0.46 | 0.26 | 0.28 | 0.026 | 0.37 | 0.12 | P>0.05 |
| Lachnospiraceae | | Oribacterium | 2.3^a^ | 0.38 | 0.82^b^ | 0.22 | 1.57 | 0.39 | P<0.05 |
| Lachnospiraceae | | UCG-009 | 0.23^a^ | 0.02 | 0.12^b^ | 0.02 | 0.17 | 0.027 | P<0.05 |
| Lachnospiraceae | | Lachnoclostridium_1 | 0.14 | 0.01 | 0.19 | 0.05 | 0.17 | 0.02 | P>0.05 |
| Lachnospiraceae | | Acetitomaculum | |  | 0.11 |  |  |  |  |
| Lachnospiraceae | | possible_genus_Sk018 | 0.15 |  |  |  |  |  |  |
| Lachnospiraceae | | Moryella |  |  | 0.07 |  |  |  |  |
| Acidaminococcaceae | | Succiniclasticum | 15 | 4.4 | 6.2 | 0.44 | 10.6 | 2.79 | P>0.05 |
| Veillonellaceae | | | 3.67 | 2.081 | 4.6 | 2.34 | 4.13 | 1.42 | P>0.05 |
| Veillonellaceae | | Schwartzia | 0.32 | 0.03 | 0.34 | 0.055 | 0.3 | 0.03 | P>0.05 |
| Veillonellaceae | | Selenomonas_1 | 3.34 | 2.08 | 4.26 | 2.29 | 3.8 | 1.4 | P>.05 |
| Family_XI | | | 0.2 | 0.02 | 0.49 | 0.13 | 0.35 | 0.08 |  |
| Family_XI | Anaerovorax | | 0.14 (1n) |  | 0.35 |  |  |  |  |
| Family_XI | Sporanaerobacter | | 0.14 |  |  |  |  |  |  |
| Family_XI | Gallicola |  |  |  | 0.06 |  |  |  |  |
| Streptococcaceae | | Streptococcus | 1.97 | 1.09 | 0.6 | 0.19 | 1.29 | 0.58 |  |
| Christensenellaceae | | R-7_group | 4.7 | 0.56 | 5.16 | 0.95 | 4.9 | 0.5 |  |
| Lactobacillaceae | | Lactobacillus | 0.2 |  | 0.1 |  |  |  |  |
| Phylum Proteobacteria | | | | | | | | | |
| Succinivibrionaceae | | Succinivibrio | 0.05 (1n) |  | 0.22 |  |  |  |  |
| Burkholderiaceae | | Burkholderia-Caballeronia-Paraburkholderia | 0.06 (1n) |  | 0.098 |  |  |  |  |
| Phylum Synergistetes | | | | | | | | | |
| Synergistaceae | | Pyramidobacter | |  | 0.16 |  |  |  |  |
