# Supplementary material for: Fibrolytic rumen bacteria of camel and sheep and their applications in the bioconversion of barley straw to soluble sugars for biofuel production
Source: PLoS One. 2022 Jan 7;17(1):e0262304. doi: 10.1371/journal.pone.0262304 (PMC8740978; doi:10.1371/journal.pone.0262304)
Supplement: S1 Fig — A comparison of the relative abundances of the main bacterial groups in camel in the current study (Camel-A) and a previous study (Camel-B), Sheep in the current study (Sheep-A) and a previous study (Sheep-B), cattle, buffalo, and deer. (PDF) [file pone.0262304.s002.pdf]

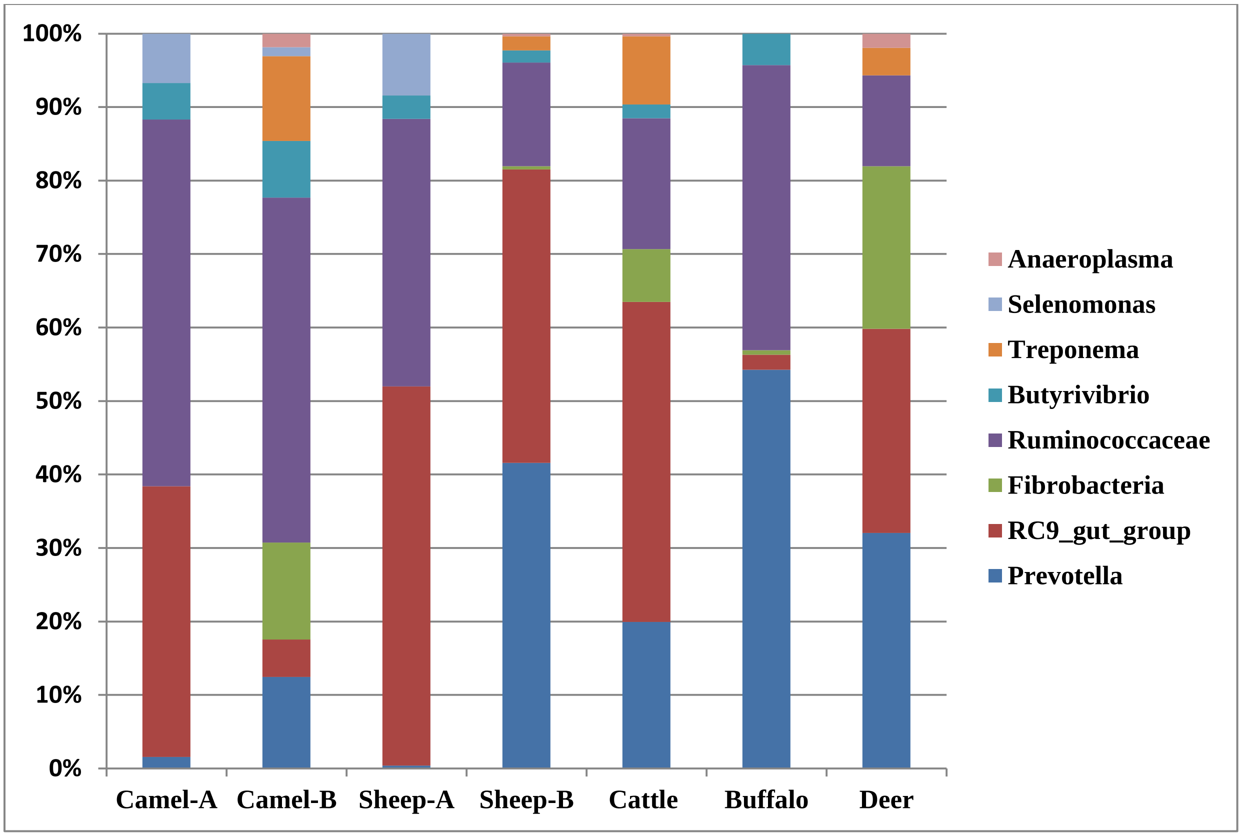

S2 Fig 1: The relative abundances of main rumen bacterial groups in different animal species: A comparison of the relative abundances of the main bacterial groups in camel in the current study (Camel-A) and a previous study (Camel-B), Sheep in the current study (Sheep-A) and a previous study (Sheep-B), cattle, buffalo, and deer.
