# Supplementary material for: Fibrolytic rumen bacteria of camel and sheep and their applications in the bioconversion of barley straw to soluble sugars for biofuel production
Source: PLoS One. 2022 Jan 7;17(1):e0262304. doi: 10.1371/journal.pone.0262304 (PMC8740978; doi:10.1371/journal.pone.0262304)
Supplement: Attachment — Submitted filename: Comments.docx [file pone.0262304.s003.docx]

I think the research is interesting.

However, was there anything novel about the functionality of the rumen microbial community in sheep and camels? was not sufficiently appealing to me.

For example, it would be good if the differences between the rumen microbial community of sheep and camels and that of other rumen microbes could be more clearly expressed in graphs.

Figure1 Electron micrographs were not clear, so it is difficult to see the microorganisms attached to the surface of straws. Change to a clearer photo? Or isn't it necessary to include a photo?

Table1 Please add the unit of gas production.

Table1 "Inoculum Source" means “samples after 72 hours incubation”, is it correct? Please have a representation of what the sample is so that everyone can understand what it was.

Table 1　 What does "overall" in Table 1 mean?

I think that DNA extraction is extracting the solid (i.e., attached to the straw), but does "overall" include not only the solid but also the cultured liquid? Is it correct to say that the term "overall" includes not only solids but also cultured liquids? Please describe this in an easy-to-understand manner.

I did not understand, why you wrote camel rumen sample to “FC”, and inoculum sheep was “FR”. Wouldn't it be better to be consistent all the time in the paper, with camel samples and sheep samples?

Table 3 please add the units for value. (%)

Line 435

This time, the barley straw is autoclaved at 120 degrees Celsius, which I think is quite a heat treatment to the straw. This is a considerable heat treatment. It is possible that the effect of this heat treatment may have increased the hydrolysis efficiency, but what do you think about this? Also, I think this will be a problem when discussing the degradation efficiency when comparing with other papers.

So, I think it would be better to have the results of how it was when it was not autoclaved.
