# Supplementary material for: Fibrolytic rumen bacteria of camel and sheep and their applications in the bioconversion of barley straw to soluble sugars for biofuel production
Source: PLoS One. 2022 Jan 7;17(1):e0262304. doi: 10.1371/journal.pone.0262304 (PMC8740978; doi:10.1371/journal.pone.0262304)
Supplement: Attachment — Submitted filename: 000-Response to Reviewers.docx [file pone.0262304.s004.docx]

Desert Research Center	23^th^ November 2021

1Mathaf El Matariya St.B.O.P.11753

Matariya- Cairo,Egypt

Phone: (+202)26332846 - 26374800

FAX: (+202) 26357858

Email: [Rabee_a_m@yahoo.com](mailto:Rabee_a_m@yahoo.com)

PlOS ONE editorial office

**Fibrolytic rumen bacteria of camel and sheep and their applications in the bioconversion of barley straw to soluble sugars for biofuel production**

We thank you and the Reviewers for their constructive comments and corrections. We responded to all comments and have extensively modified many sections in the paper to correct grammatical and style errors. I also enclosed unclean paper including all comments colored by yellow. Below are the responses to all the comments. We made a slight modification in the title of the manuscript. Our paper was improved greatly after the response to the reviewers’ comments.

We appreciate the opportunity to submit our manuscript to PlOS ONE.

Yours sincerely,

Dr. Alaa Rabee

Researcher at Desert Research Center, Egypt

On behalf of all authors

**Comments**

Manuscript title: **“Fibrolytic rumen bacteria of camel and sheep and their applications in the bioconversion of barley straw to soluble sugars for biofuel production”**

In the unclean or marked manuscript you could notice that colored comments using yellow.

**Responses**

**I think the research is interesting. However, was there anything novel about the functionality of the rumen microbial community in sheep and camels? was not sufficiently appealing to me. For example, it would be good if the differences between the rumen microbial community of sheep and camels and that of other rumen microbes could be more clearly expressed in graphs.**

>> Thank so much, we added a small comparison at the end of discussion and added supplementary figure that compare the relative abundances of main bacterial groups in our study and in the rumen of other ruminant animals.

**Figure1 Electron micrographs were not clear, so it is difficult to see the microorganisms attached to the surface of straws. Change to a clearer photo? Or isn't it necessary to include a photo?**

>>We removed it, unfortunately we don’t have another photos. We had to take photos at different incubation times before 72 h to get clear photos for bacterial colonization.

**Table1 Please add the unit of gas production.**

>> Thank you, we added it and scanned all tables for similar mistaks.

**Table1 "Inoculum Source" means “samples after 72 hours incubation”, is it correct? Please have a representation of what the sample is so that everyone can understand what it was.**

>> Thank you, inoculum Source refers to animal source of rumen samples that were inoculated into barley straw plus bacterial media, camel or sheep; we clarified that throughout the manuscript.

**Table 1　What does "overall" in Table 1 mean?**

**I think that DNA extraction is extracting the solid (i.e., attached to the straw), but does "overall" include not only the solid but also the cultured liquid? Is it correct to say that the term "overall" includes not only solids but also cultured liquids? Please describe this in an easy-to-understand manner.**

>> Thank you for this valuable comment. Overall refers to overall mean of the values in camels and sheep (6 samples: 3 for sheep and 3 for camels), we clarified that in all tables.

**I did not understand, why you wrote camel rumen sample to “FC”, and inoculum sheep was “FR”. Wouldn't it be better to be consistent all the time in the paper, with camel samples and sheep samples?**

>> Thank you, we modified that in the text and tables. We stuck with inoculum instead of sample throughout the manuscript, we modified that.

**Tables 3 please add the units for value. (%).**

>> We added it and scanned the tables for similar mistakes.

Line 435 : **This time, the barley straw is autoclaved at 120 degrees Celsius, which I think is quite a heat treatment to the straw. This is a considerable heat treatment. It is possible that the effect of this heat treatment may have increased the hydrolysis efficiency, but what do you think about this? Also, I think this will be a problem when discussing the degradation efficiency when comparing with other papers. So, I think it would be better to have the results of how it was when it was not autoclaved.**

>>> Thank you for this valuable comment. We used three serum bottles (medium + barley straw) without inoculation as blanks to allow the correction for dry matter disappearance during sterilization. However, I support your view; the microbial hydrolysis efficiency could be increased compared to the results of other studies. Therefore, we modified that section.
